# Three-dimensional digital anatomical measurement of modified sacroiliac screws

**DOI:** 10.1186/s13018-022-03018-3

**Published:** 2022-03-04

**Authors:** Tingguang Wang, Bei Zhao, Jun Yan, Jia Wang, Chong Chen, Weidong Mu

**Affiliations:** 1 Department of Traumatic Orthopaedics, Shandong Provincial Hospital, Cheeloo College of Medicine, Shandong University, 324 Jing Wu Road, Jinan, 250012 Shandong China; 2 Department of Orthopaedics, People’s Hospital of Zouping City, Binzhou, Shandong China; 3 Department of Orthopaedics, Liaocheng People’s Hospital, Liaocheng, Shandong China

**Keywords:** Modified sacral-iliac screw, Three-dimensional, Reconstruction, Screw parameters

## Abstract

**Purpose:**

To rebuild a model of the pelvis and effectively simulate the trajectory of modified sacroiliac screws, we measured the parameters of each screw and screw channel and assessed the safety and feasibility of the parameters in adults.

**Method and materials:**

One hundred (50 males and 50 females) normal adult pelvic computed tomography (CT) images were randomly selected and imported into Mimics software to rebuild the three-dimensional pelvis model. In these models, each ideal channel of modified screws was simulated, and then we obtained the precise parameters of screws and channels using Mimics and Three-matic software.

**Results:**

The results of the comparison (right vs. left) showed that there were no significant differences in any of the angles, radius or M1SI parameters (the first modified sacroiliac). However, one parameter (BS) of M2SI (the second modified sacroiliac), two parameters (AP and BS) of M3SI (the third modified sacroiliac), and three parameters (AP, BS, L) of M4SI (the fourth modified sacroiliac) were statistically significant (*P* < 0.05). The result of comparison (between genders) showed that there were no significant differences in M1SI and M2SI; in contrast, the radius, length and the *α* angle of M3SI and M4SI were significantly different between genders (*P* < 0.05), and the radius of M4SI required special attention. If the radius of the limiting screw channel was > 3.50 mm, 52 cases (52%, 24 males and 28 females) could not complete the M4SI screw placement among 100 samples. If the radius of the limiting screw channel was > 3.0 mm, a total of 10 cases (10%, 2 males and 8 females) could not complete the M4SI screw placement.

**Conclusion:**

Through the measurement of 100 healthy adult real three-dimensional pelvic models, we obtained the parameters of each modified sacroiliac screw and measured the three angles of each screw based on international coordinates for the first time, which can instruct clinical application.

## Introduction

In modern society, high-energy traumatic injuries leading to sacroiliac joint dislocation, sacral alar fracture, and loss of posterior pelvic ring integrity are common [1]. At present, there are various fixation methods for the posterior pelvic ring and fracture of the local region, such as traditional sacroiliac screw fixation, anterior or posterior sacroiliac joint plate fixation, posterior tension band plate fixation of the sacrum, and sacropelvic fixation based on screw rods [2]. Although there are a variety of fixation methods, some patients experience lumbosacral pain, internal fixation fracture, fracture end displacement again and other sequelae of different degrees [3]. Therefore, it is imperative to choose a simple and effective flexible fixation method.

In 2007, Speller et al. first reported that S2AI (the second sacral alar-iliac) screws were successfully used for the first long-stage spinal fusion in paediatric patients [4, 5]. A meta-analysis [5] of iliac screw versus S2 alar-iliac screw fixation in adults showed that compared with iliac screw (IS), the S2AI technique has a lower rate of incision infection, revision for device failure, and instrumentation-related pain rate. Another article [6] reported that with a 75% reduction in complications compared to the IS (iliac screw) technique, S2AI also has the advantage of reducing skin and soft tissue dissection, obviating the offset connector [4], and thus reducing the amount of incision bleeding, wound healing problems and other adverse factors. In 2013, Dr. Mattei et al. [7] reported a technique that combined S1AI (the first sacral alar-iliac) and S2AI screws as a salvage procedure for pseudarthrosis in the lumbar-sacral junction. In 2020, it was first reported that S3AI (the third sacral alar-iliac) screws were successfully used for lumbo-sacral fusion [4]. The above fixation methods originated in the Galveston technique, which was first proposed by Allen and Ferguson in 1984 [8], so we called it the optimized Galveston technique and used it to treat flat-back syndrome and kyphosis, pelvic obliquity, high-grade spondylolisthesis, neuromuscular scoliosis, early-onset scoliosis and so on [7, 9–13]. The key to the success of SAI technology lies in the SAI screw fixation method.

In view of the SAI screw advantage, we tried to place the traditional sacroiliac screw based on the SAI screw trajectory [14], and we called these screw-modified sacroiliac screws, which changed the traditional sacroiliac screw conventional channel. The traditional sacroiliac screw was inserted into the sacrum through the iliac bone, which was surrounded by cancellous bone, reducing the screw holding force and possibly damaging important neurovascular structures in the pelvis, which can have deleterious consequences [15]. There have been few reports on the modified sacroiliac screw and trajectory in previous studies; therefore, we selected the pelvic CT data of 100 healthy adults to reconstruct each pelvic model and simulate the placement of modified sacroiliac screws with software. The screw parameters of each modified sacroiliac screw were measured to provide accurate reference data for clinical applications. To our knowledge, regarding either parameter measurement or comparison, our sample size is the largest series to date in this field.

## Materials and methods

### Subjects

After approval from our institutional review board, we randomly selected 100 adult patients who had normal pelvic computed tomography (CT) scans in our hospital from the beginning of January 2020 to the end of August 2021. In our institution’s Picture Archiving and Communication Systems of patients, “pelvis” was retrieved as a keyword, and about 300 normal pelvic data were randomly selected based on the report of no abnormal. We used a systematic random sampling method of selecting one in three to obtain 100 samples from 300 patients. The initial medical indications include: a. Injuries caused by traffic accident, fall accident and so on; b. Pain due to lumbosacral or pelvic area; c. Physical check-up. The inclusion criteria include: a. The normal adult pelvis; b. Correct body posture during CT examination; c. Age limit between 18 and 80 years. The exclusion criteria include: a. Pelvic malformations; b. History of pelvic fractures and surgery; c. Bone metabolic diseases; d. Long-term use of hormones and anticoagulants. Our study population consisted of 100 patients (50 males and 50 females) with a mean age and standard deviation of 42.32 ± 16.53 years (range 18–76 years).

We further examined the selected patients according to the inclusion criteria. Due to the retrospective nature of the study, informed consent was waived.

### Three-dimensional reconstruction of pelvis

Data from each individual were imported into Mimics (Materialise, Belgium; version 20.0) software in DICOM (Digital Imaging and Communications in Medicine) format, and then coronal, sagittal and axial images were obtained. Mimics “thresholding” and “region growing” functions were used to form a mask of the sacrum and two ilium bones. The “calculate part” function was used to generate a rough three-dimensional model of the pelvis. Then, the model was subjected to “smooth” and “wrap” operations to obtain a smooth three-dimensional pelvic model from real data for future use.

### Simulation process and illustration

**Fig. 1:**
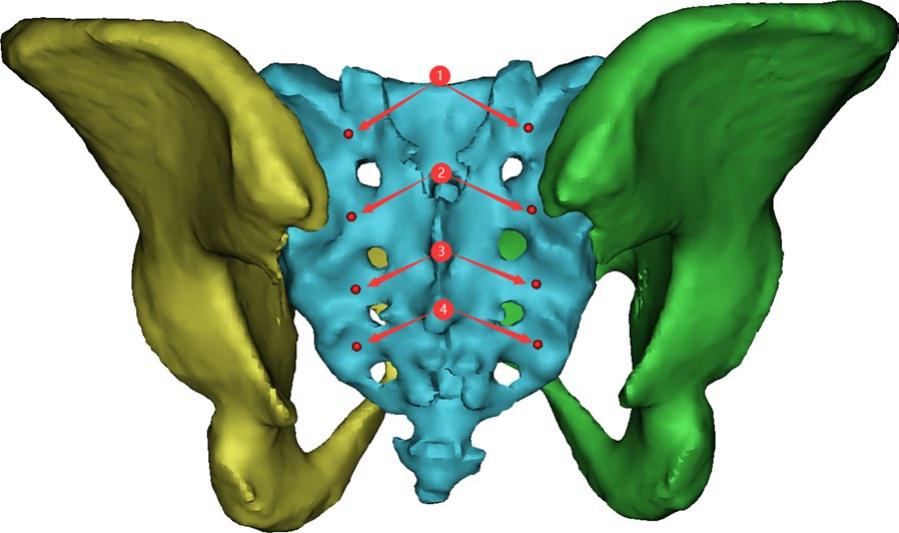
Importing the data and reconstructing the pelvic model. The sacral entry point for each modified sacroiliac screw was also marked according to previous literature

**Fig. 2:**
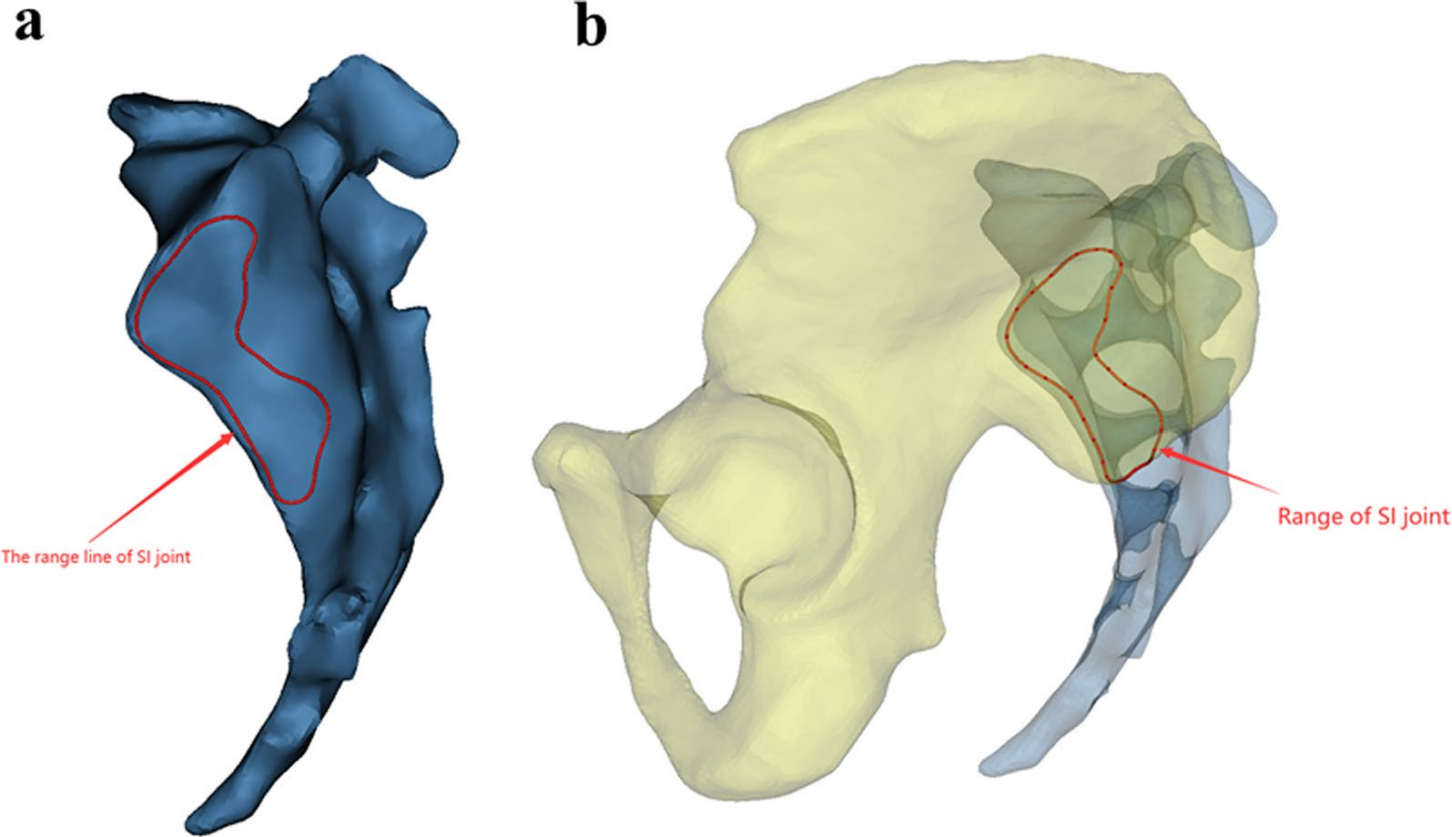
**a**, **b** To ensure that each modified sacroiliac screw channel was within the range of the sacroiliac joint, the range of the sacroiliac joint was marked. The contralateral method was consistent

**Fig. 3:**
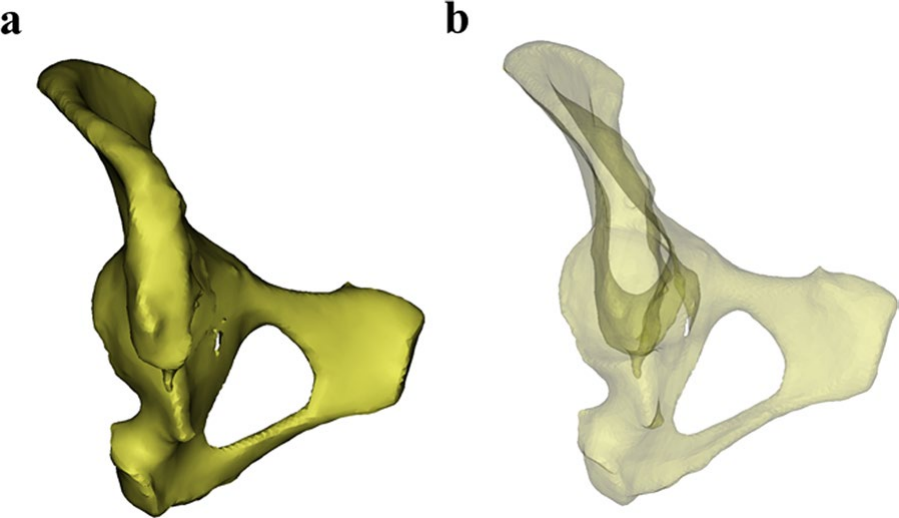
**a**, **b** The transparency function of the software was used to make the ilium transparent and rotate the ilium until the appearance of the “tear drop” was completely seen. The tear drop was the channel filled with cancellous bone between the medial and lateral cortical bone of the ilium

**Fig. 4:**
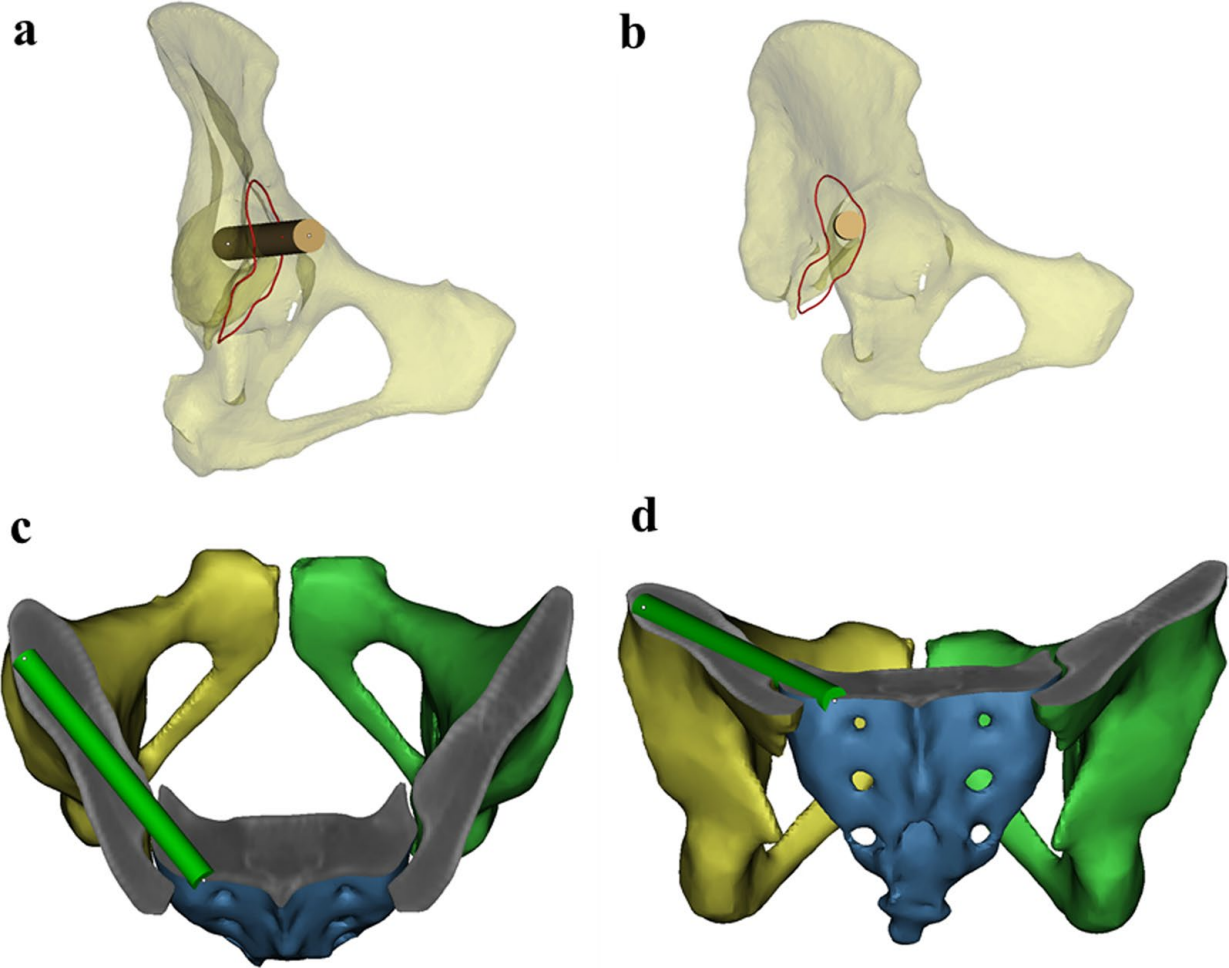
**a**, **b** The cylinder was resized to reach the maximum value of the radius and we confirmed that it was within the range of the teardrop and sacroiliac joint; **c**, **d** Display of the full length of the cylinder, further confirming that it did not cause violation of the medial and lateral cortical bone of the ilium

**Fig. 5:**
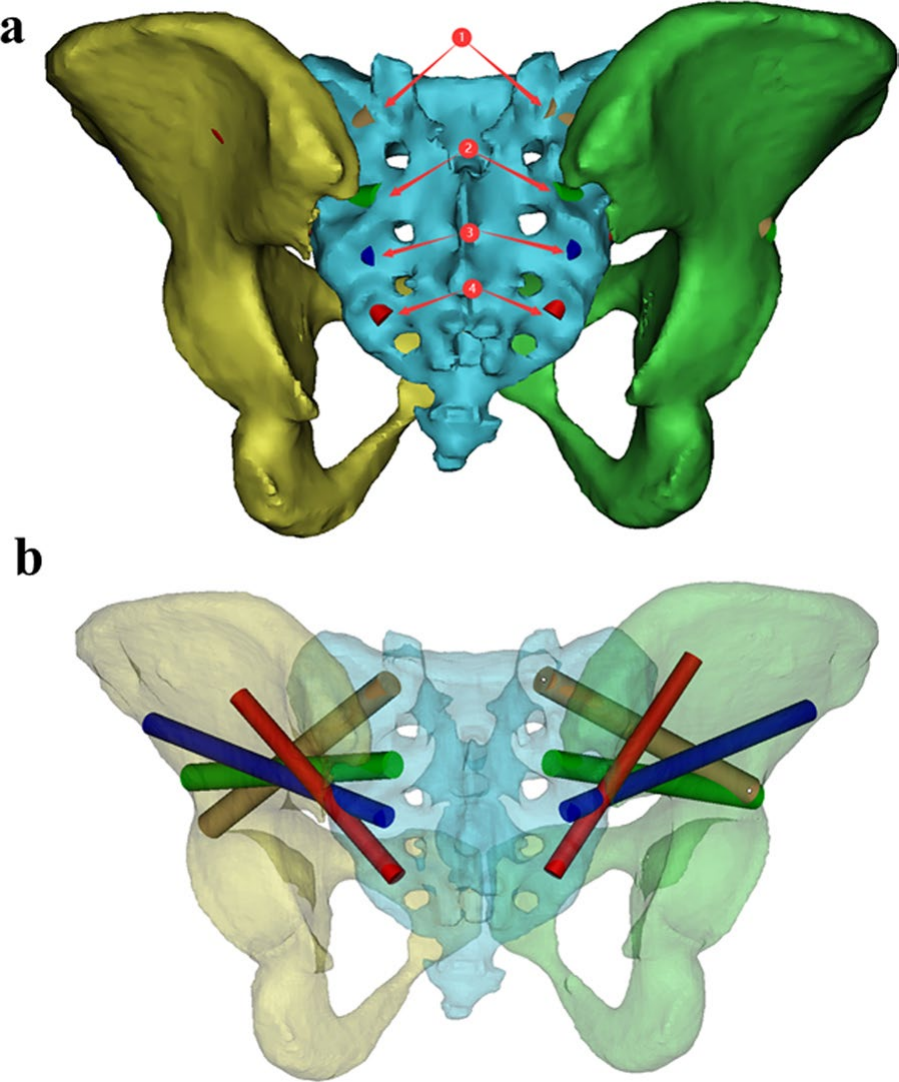
**a** According to the operating requirements of Fig. 4, four modified sacroiliac screws were placed on the left and right sides of the sacrum; **b** The position and orientation of each screw were shown by a transparent pelvic model

To ensure the authenticity and reliability of the screw parameters, the operating procedures and steps we used were consistent for each pelvic model. First, the data were imported, and the pelvic model was reconstructed using commands such as “CT Bone”, “Calculate”, “wrap” and “smooth” in Mimics software. The sacral entry point for each screw was also marked according to previous literature (Fig. 1). Second, the sacrum and ilium were hidden, the sacroiliac joint characterized by the “auricular plane” of the sacrum was exposed, and the range of the sacroiliac joint was marked with the function of “LINE” and “Point” (Fig. 2). Third, the transparency function of the software was used to make the ilium transparent and rotate the ilium until the appearance of the “tear drop” was completely seen (Fig. 3). Next, the modified sacroiliac screw trajectory began at the entry point (as shown in Fig. 1) on the dorsal side of the sacrum, passed through the sacral alar and sacroiliac joint, and extended into the iliac wing. The model was made transparent to ensure that this screw channel was within the marked “tear drop” range of the ilium and sacroiliac joint, and then it was resized to reach the maximum value of the radius and length, without causing the violation of the medial and lateral cortical bone of the ilium and the range of the protruding sacroiliac joint (Fig. 4). Finally, all modified sacroiliac screw channels were simulated and reconstructed based on the above steps (Fig. 5).

### Measurement process

**Fig. 6:**
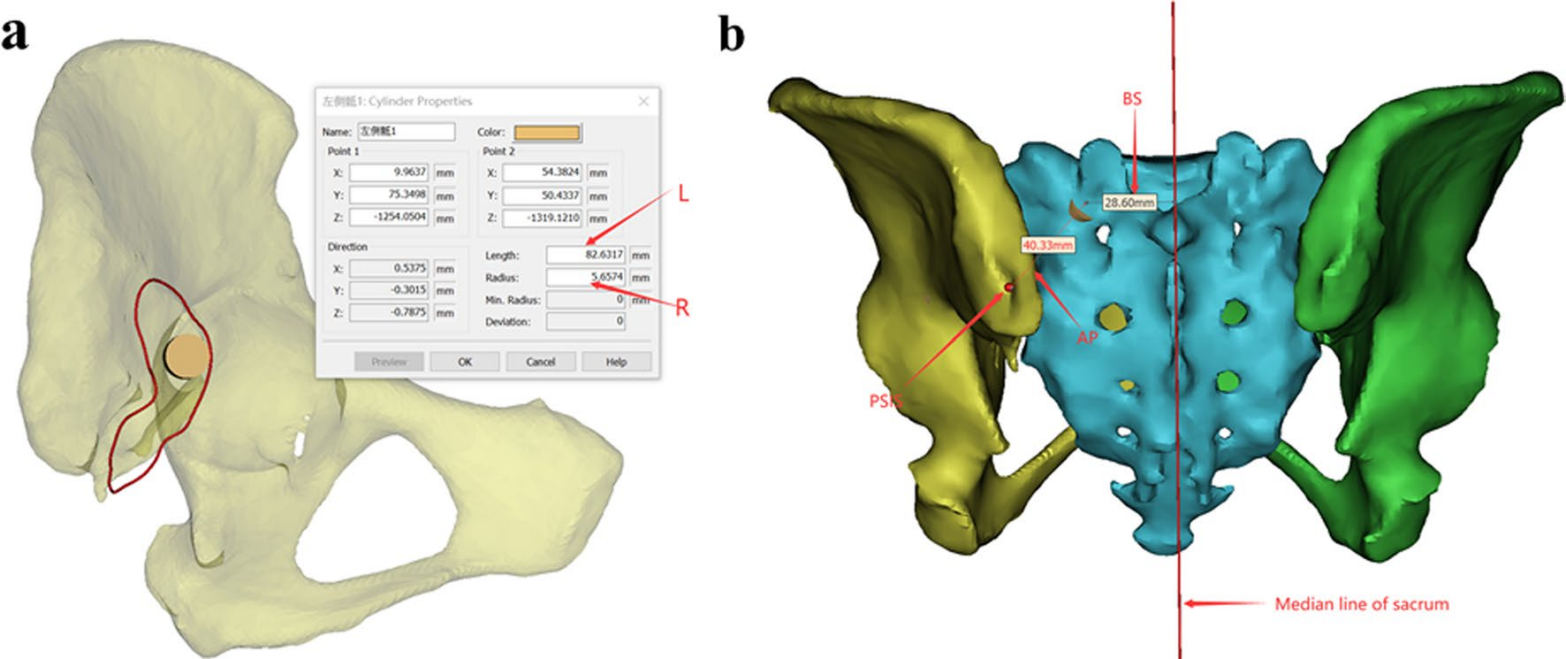
**a** According to our requirements, the maximum screw radius and the maximum screw length were obtained by reading the cylinder properties after ideal screw placement; **b** The posterior superior iliac crest and the sacral midline on one side were marked, and the distance to the posterosuperior iliac crest and the sacral midline was measured using the centre of the cylinder as the starting point. The contralateral method was consistent

**Fig. 7:**
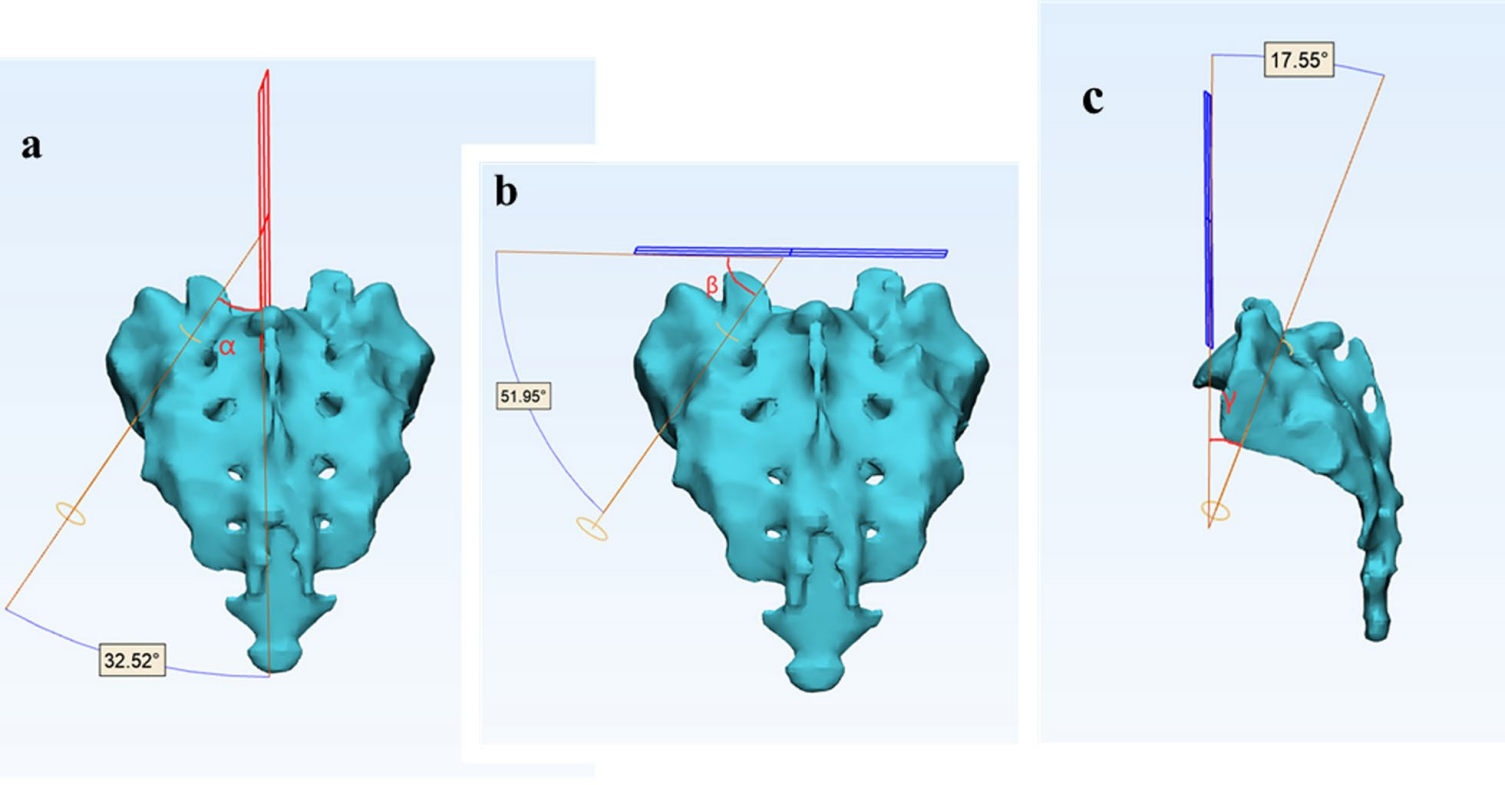
**a**: *α* angle: angle between axis of modified sacroiliac screw and sagittal plane; **b**: *β* angle: angle between axis of modified sacroiliac screw and transverse plane; **c**: *γ* angle: angle between axis of modified sacroiliac screw and coronal plane

The measurement process was divided into two steps. First, the radius and length of the screw were obtained by checking the properties of the screw in the Mimics software, and the distance from the screw entry point to the posterior superior iliac crest and the sacral midline was obtained by measuring the length (Fig. 6). Second, using Three-matic software, the sagittal plane, transverse plane and coronal plane were established based on the world coordinates. The angle between the screw trajectory and sagittal plane was the *α* angle. The angle between the screw trajectory and transverse plane was the *β* angle. The angle between the screw trajectory and coronal plane was the *γ* angle. The three angles were measured by measuring the angle between the line and plane in three matrices (Fig. 7).

### Statistical analysis

Data were presented as the mean ± standard deviation. If variance between the case and control groups was equal, Student’s t test or paired t test was used to compare the difference of means of continuous variables. Otherwise, the Wilcoxon rank sum test or signed-rank test was used. All analyses were carried out using SPSS (Statistical Product and Service Solutions version 25.0). All reported probabilities (*P* value) were two sided. *P* < 0.05 was considered as statistically significant.

## Results

We obtained accurate data for each modified sacroiliac screw using Mimics and Three-matic software, including the radius, length, positioning distance, and three angles. We conducted a comparison between males and females, for the right and left sides, with SPSS (Statistical Product and Service Solutions version 25.0) and obtained the following results:

**1.** The parameter ranges of the M1SI (the first modified sacroiliac) screws were presented as the mean ± standard deviation. The range of the radius of the screw was 4.92 ± 0.36 mm to 4.99 ± 0.37 mm; the range of the length of the screw was 100.87 ± 13.15 mm to 103.39 ± 12.89 mm; the range of the distance between the entry point and the PSIS (the Posterior Superior Iliac Spine) was 35.47 ± 6.60 mm to 37.02 ± 6.21 mm; the range of the distance between the entry point and the median sacral crest was 30.22 ± 3.86 mm to 31.31 ± 4.58 mm; the range of the *α* angle was 37.05° ± 4.62° to 37.94° ± 4.64°; the range of the *β* angle was 43.01° ± 6.25° to 44.21° ± 6.04°; and the range of the *γ* angle was 21.27° ± 5.56° to 22.23° ± 6.72°.

As summarized in Tables 1 and 2, there was no significant difference in any parameters of the first modified sacroiliac screw, either between left and right or between sexes (*P* > 0.05).

**Table 1:** M1SI screw parameters right and left comparison

Parameter	Mean ± SD		Comparison (Right vs Left)	
	Right (*n* = 100)	Left (*n* = 100)	Statistical value	*P* value
R (mm)	4.96 ± 0.38	4.96 ± 0.36	0.78^※^	0.44
L (mm)	101.98 ± 12.85	102.13 ± 13.37	0.28^#^	0.78
AP (mm)	36.48 ± 6.27	36.24 ± 6.42	0.82^#^	0.41
BS (mm)	30.17 ± 3.96	30.77 ± 4.25	1.61^#^	0.11
*α* (°)	37.42° ± 4.58°	37.91° ± 4.67°	0.84^#^	0.41
*β* (°)	43.69° ± 6.40°	43.48° ± 5.92°	0.42^#^	0.68
*γ* (°)	22.1° ± 6.66°	21.75° ± 6.59°	0.66^#^	0.51

※ Wilcoxon signed-rank test; # paired t test

**Table 2:** Comparison of M1SI screw parameters between genders

Parameter	Right (Mean ± SD)				Left (Mean ± SD)			
	Male (*n* = 50)	Female (*n* = 50)	Statistical value	*P* value	Male (*n* = 50)	Female (*n* = 50)	Statistical value	*P* value
R (mm)	4.98 ± 0.38	4.94 ± 0.38	0.81^※^	0.42	4.99 ± 0.37	4.92 ± 0.36	1.19^※^	0.23
L (mm)	103.08 ± 13.17	100.90 ± 12.57	0.85^#^	0.39	103.39 ± 12.89	100.87 ± 13.85	0.94^#^	0.35
AP (mm)	37.01 ± 6.12	35.95 ± 6.43	0.85^#^	0.39	37.02 ± 6.21	35.47 ± 6.60	1.21^#^	0.23
BS (mm)	30.74 ± 3.76	29.59 ± 4.11	1.47^#^	0.15	31.31 ± 4.58	30.22 ± 3.86	1.29^#^	0.19
*α* (°)	37.78° ± 3.55°	37.05° ± 4.62°	0.79^#^	0.43	37.88° ± 4.74°	37.94° ± 4.64°	0.06^#^	0.95
*β* (°)	43.17° ± 6.77°	44.21° ± 6.04°	0.81^#^	0.42	43.01° ± 6.25°	43.96° ± 5.59°	0.81^#^	0.42
*γ* (°)	22.23° ± 6.72°	21.97° ± 6.66°	0.19^#^	0.85	22.22° ± 7.50°	21.27° ± 5.56°	0.92^※^	0.36

※Two-sample Wilcoxon rank-sum (Mann–Whitney) test; #two-sample t test with equal variances

**2.** The parameter ranges of the M2SI (the second modified sacroiliac) screw were presented as the mean ± standard deviation. The range of the radius of the screw was 4.65 ± 0.34 mm to 4.71 ± 0.29 mm; the range of the length of the screw was 109.89 ± 5.98 mm to 112.59 ± 7.67 mm; the range of the distance between the entry point and the PSIS (the Posterior Superior Iliac Spine) was 24.92 ± 3.81 mm to 25.65 ± 5.14 mm; the range of the distance between the entry point and the median sacral crest was 28.27 ± 4.09 mm to 29.92 ± 3.19 mm; the range of the *α* angle was 32.99° ± 5.03° to 39.77° ± 4.97°; the range of the *β* angle was 24.95° ± 6.43° to 25.33° ± 5.72°; and the range of the *γ* angle was 39.37° ± 4.82° to 40.29° ± 5.65°.

As summarized in Tables 3 and 4, there were significant differences in BS (the distance from the entry point to the median sacral crest) (*P* < 0.01) in the left–right comparison, and other parameters had no significant difference between the left–right comparison and sexes (*P* > 0.05).

**Table 3:** M2SI screw parameters right and left comparison

Parameter	Mean ± SD		Comparison (Right vs Left)	
	Right (*n* = 100)	Left (*n* = 100)	Statistical value	*P* value
R (mm)	4.68 ± 0.32	4.69 ± 0.3	1.29※	0.19
L (mm)	111.24 ± 6.97	111.04 ± 7.44	0.54#	0.59
AP (mm)	25.58 ± 4.56	25.29 ± 4.52	0.99#	0.33
BS (mm)	28.49 ± 4.06	29.68 ± 3.49	4.29#	< 0.01
*α* (°)	38.91° ± 4.48°	39.3° ± 4.55°	0.84※	0.41
*β* (°)	25.2° ± 5.67°	25° ± 6.08°	0.48#	0.63
*γ* (°)	40.08° ± 5.93°	39.83° ± 5.24°	0.40#	0.69

※ Wilcoxon signed-rank test; # paired t test

**Table 4:** Comparison of M2SI screw parameters between genders

Parameter	Right (Mean ± SD)				Left (Mean ± SD)			
	Male (*n* = 50)	Female (*n* = 50)	Statistical value	*P* value	Male (*n* = 50)	Female (*n* = 50)	Statistical value	*P* value
R (mm)	4.71 ± 0.29	4.65 ± 0.34	0.43※	0.66	4.71 ± 0.29	4.67 ± 0.31	0.41※	0.69
L (mm)	112.59 ± 7.67	109.89 ± 5.98	1.96#	0.06	112.14 ± 7.70	109.95 ± 7.07	0.35#	1.21
AP (mm)	25.62 ± 5.04	25.53 ± 4.07	0.11#	0.92	25.65 ± 5.14	24.92 ± 3.81	0.77※	0.44
BS (mm)	28.27 ± 4.09	28.71 ± 4.07	0.54#	0.59	29.43 ± 3.78	29.92 ± 3.19	0.71#	0.49
*α* (°)	32.99° ± 5.03°	34.98° ± 5.44°	0.27#	0.78	38.84° ± 4.09°	39.77° ± 4.97°	0.94※	0.35
*β* (°)	25.07° ± 5.68°	25.33° ± 5.72°	0.23#	0.82	25.06° ± 5.77°	24.95° ± 6.43°	0.09#	0.93
*γ* (°)	40.02° ± 6.36°	40.14° ± 5.52°	0.11#	0.92	40.29° ± 5.65°	39.37° ± 4.82°	0.88#	0.38

※Two-sample Wilcoxon rank-sum (Mann–Whitney) test; #two-sample t test with equal variances

**3.** The parameter ranges of the M3SI (the third modified sacroiliac) screw were presented as the mean ± standard deviation. The range of the radius of the screw was 3.91 ± 0.08 mm to 4.26 ± 0.44 mm; the range of the length of the screw was 120.56 ± 15.45 mm to 128.05 ± 8.15 mm; the range of the distance between the entry point and the PSIS (the Posterior Superior Iliac Spine) was 32.45 ± 5.68 mm to 34.58 ± 6.39 mm; the range of the distance between the entry point and the median sacral crest was 27.73 ± 2.92 mm to 28.75 ± 3.59 mm; the range of the *α* angle was 38.06° ± 5.38° to 40.32° ± 4.41°; the range of the *β* angle was 6.16° ± 4.48° to 7.21° ± 4.77°; and the range of the *γ* angle was 48.26° ± 4.96° to 50.69° ± 5.19°.

As summarized in Tables 5 and 6, there were multiple groups of parameters with statistical significance. As shown in Table 5, there were significant differences (*P* < 0.05) between AP (the distance from the entry point to the posterior superior iliac spine) and BS (the distance from the entry point to the median sacral crest) of the third modified sacroiliac screw in the left–right comparison. As shown in Table 6, there were significant differences (*P* < 0.01) in the radius and length of the third screw between males and females, while other parameters showed no significant differences (*P* > 0.05).

**Table 5:** M3SI Screw parameters right and left comparison

Parameter	Mean ± SD		Comparison (Right vs Left)	
	Right (*n* = 100)	Left (*n* = 100)	Statistical value	*P* value
R (mm)	4.07 ± 0.51	4.08 ± 0.55	1.81※	0.71
L (mm)	124.64 ± 12.35	124.24 ± 12.64	0.88#	0.38
AP (mm)	33.99 ± 6.33	33.43 ± 5.99	2.03#	0.04
BS (mm)	27.75 ± 3.33	28.69 ± 3.35	3.63#	< 0.01
*α* (°)	38.44° ± 4.75°	39.48° ± 4.52°	1.51#	0.14
*β* (°)	6.92° ± 4.89°	6.63° ± 4.6°	1.09※	0.27
*γ* (°)	50.25° ± 4.7°	49.18° ± 4.78°	1.55#	0.12

※ Wilcoxon signed-rank test; # paired t test

**Table 6:** Comparison of M3SI screw parameters between genders

Parameter	Right (Mean ± SD)				Left (Mean ± SD)			
	Male (*n* = 50)	Female (*n* = 50)	Statistical value	*P* value	Male (*n* = 50)	Female (*n* = 50)	Statistical value	*P* value
R (mm)	4.24 ± 0.41	3.91 ± 0.08	3.42※	< 0.01	4.26 ± 0.44	3.91 ± 0.59	3.38※	< 0.01
L (mm)	128.05 ± 8.15	121.24 ± 14.77	2.73※	< 0.01	127.92 ± 7.51	120.56 ± 15.45	3.08※	< 0.01
AP (mm)	34.58 ± 6.39	33.39 ± 6.27	0.94#	0.35	34.41 ± 6.19	32.45 ± 5.68	1.65※	0.11
BS (mm)	27.73 ± 2.92	27.76 ± 3.73	0.04#	0.96	28.75 ± 3.59	28.62 ± 3.12	0.19#	0.85
*α* (°)	38.06° ± 5.38°	38.81° ± 4.04°	1.07※	0.29	38.63° ± 4.5	40.32° ± 4.41°	1.91#	0.06
*β* (°)	6.63° ± 5.03°	7.21° ± 4.77°	0.73※	0.47	7.11° ± 4.71°	6.16° ± 4.48°	1.08※	0.28
*γ* (°)	50.69° ± 5.19°	49.81° ± 4.16°	0.93#	0.35	50.09° ± 4.46°	48.26° ± 4.96°	1.94#	0.06

※Two-sample Wilcoxon rank-sum (Mann–Whitney) test; #two-sample t test with equal variances

**4.** The parameter ranges of the M4SI (the fourth modified sacroiliac) screw were presented as the mean ± standard deviation. The range of the radius of the screw was 3.29 ± 0.42 mm to 3.52 ± 0.32 mm; the range of the length of the screw was 88.63 ± 8.25 mm to 91.39 ± 8.69 mm; the range of the distance between the entry point and the PSIS (the Posterior Superior Iliac Spine) was 46.76 ± 7.37 mm to 50.90 ± 7.49 mm; the range of the distance between the entry point and the median sacral crest was 25.83 ± 3.22 mm to 27.73 ± 3.25 mm; the range of the *α* angle was 32.99° ± 5.03° to 36.13° ± 4.56°; the range of the *β* angle was 21.51° ± 8.21° to 25.07° ± 8.87°; and the range of the *γ* angle was 45.04° ± 5.55° to 45.34° ± 6.89°.

As summarized in Table 7, the length, AP (the distance from the entry point to the posterior superior iliac spine) and BS (the distance from the entry point to the median sacral crest) of the screws were significantly different (*P* < 0.05) in the comparison of left and right screw parameters. In Table 8, the radius, length, AP (the distance from the entry point to the posterior superior iliac spine) and *α* angle (angle between axis of modified sacroiliac screw and sagittal plane) of the fourth modified sacroiliac screw were significantly different (*P* < 0.05), and the other parameters showed no significant differences (*P* > 0.05). At the same time, we also found that 10 patients (10%, 2 males and 8 females) could not place the fourth modified sacroiliac screw if the radius of the screw was 3.0 mm, and 52 patients (52%, 24 males and 28 females) could not place the fourth modified sacroiliac screw if the radius of the screw was 3.5 mm.

**Table 7:** M4SI screw parameters right and left comparison

Parameter	Mean ± SD		Comparison (Right vs Left)	
	Right (*n* = 100)	Left (*n* = 100)	Statistical value	*P* value
R (mm)	3.43 ± 0.32	3.4 ± 0.39	0.41※	0.68
L (mm)	89.05 ± 9.48	90 ± 8.54	2.81#	< 0.01
AP (mm)	49.49 ± 7.43	44.83 ± 7.44	2.08#	0.04
BS (mm)	26.37 ± 3.11	27.42 ± 3.09	3.52#	< 0.01
*α* (°)	33.97° ± 5.31°	35.17° ± 4.85°	1.78#	0.08
*β* (°)	23.95° ± 8.71°	22.92° ± 8.53°	1.89#	0.06
*γ* (°)	45.29° ± 6.45°	45.1° ± 5.43°	0.28#	0.78

※Wilcoxon signed-rank test; # paired t test

**Table 8:** Comparison of M4SI screw parameters between genders

Parameter	Right (Mean ± SD)				Left (Mean ± SD)			
	Male (*n* = 50)	Female (*n* = 50)	Statistical value	*P* value	Male (*n* = 50)	Female (*n* = 50)	Statistical value	*P* value
R (mm)	3.51 ± 0.29	3.35 ± 0.33	2.49#	0.01	3.52 ± 0.32	3.29 ± 0.42	2.01※	0.04
L (mm)	89.32 ± 9.77	88.78 ± 9.26	2.17※	0.03	91.39 ± 8.69	88.63 ± 8.25	2.24※	0.03
AP (mm)	50.90 ± 7.49	48.08 ± 7.18	2.06※	0.04	50.89 ± 6.98	46.76 ± 7.37	2.87#	< 0.01
BS (mm)	26.90 ± 2.92	25.83 ± 3.22	1.74#	0.09	27.73 ± 3.25	27.11 ± 2.91	1.01#	0.31
*α* (°)	32.99° ± 5.03°	34.98° ± 5.44°	1.89#	0.04	34.21° ± 4.98°	36.13° ± 4.56°	2.01#	0.04
*β* (°)	25.07° ± 8.87°	22.83° ± 8.48°	1.29#	0.19	24.33° ± 8.66°	21.51° ± 8.21°	1.67#	0.09
*γ* (°)	45.34° ± 6.89°	45.24° ± 6.04°	0.07#	0.94	45.16° ± 5.37°	45.04° ± 5.55°	0.11#	0.92

※Two-sample Wilcoxon rank-sum (Mann–Whitney) test; #two-sample t test with equal variances

## Discussion

With the acceleration of the rhythm of life and the different travel modes in modern society, various high-energy injuries such as car accident injuries, high-energy blunt injuries and fracture injuries caused by falling from a height have become more common. In particular, pelvic fracture and traumatic spinopelvic dissociation cause the most serious consequences, ranging from physical dysfunction or disability to life-threatening conditions [16, 17]. Although the design of medical equipment and the development of various technical methods for spinal and pelvic surgery internal fixation have made many new advances, achieving stable sacral pelvic fixation is still one of the most difficult challenges in orthopaedic surgery [15, 18]. There are two reasons for this challenge. First, the lumbosacral region is characterized by a unique complex anatomical structure, and, second, current devices for the treatment of fractures do not perfectly solve the problem of fracture internal fixation. At present, there are various fixation methods for the posterior pelvic ring and fracture of the local region, such as the traditional sacroiliac screw, anterior or posterior sacroiliac joint plate fixation, posterior tension band plate fixation of the sacrum, and sacropelvic fixation based on screw-rod, etc. In numerous fixation devices, sacroiliac screws have been applied by clinicians, because of their advantages of minimal invasiveness, a short learning curve and easy placement. Hence, instead of being phased out, these screws have been greatly developed in recent years, with advancements ranging from simple single sacroiliac screw fixation to percutaneous cement sacroiliac screw fixation [19–21] and the recent emergence of lengthening sacroiliac screws through the bilateral sacroiliac joint [22]. Despite the development of sacroiliac screws, many problems caused by the sacroiliac screw itself cannot be changed, such as screw fracture, insufficient internal control force, poor stability, and important neurovascular injury in the screw placement process. However, the development of sacroiliac screws reflects the idea that clinicians want to explore and implement a reliable, fixed and effective screw.

Lumbar-sacral fusion has been utilized in many clinical scenarios, such as flat-back syndrome and kyphosis, pelvic obliquity, high-grade spondylolisthesis, and extensive sacropelvic tumour resection [7, 9–13]. Lumbar-sacral fusion surgery has experienced approximately three generations of development. The first-generation technique was the Galveston technique in 1984, the second-generation technique involved iliac screws with connectors, and the third-generation technique was the sacral alar-iliac (SAI) screw technique. The first S2AI screw was successfully performed in paediatric spinal surgery by Dr. Sponseller and Dr. Kebaish in 2007 [23, 24]. Dr. Mattei et al. reported the combined use of S1AI and S2AI as a remedy and expanded the scope of application of the SAI technique in 2013 [1]. S3AI was first reported as a new long-stage spinal fusion anchor point in 2020 [2]. The SAI technique had several strengths: improved construct stability and biomechanical torsion and reduced complications, including implant prominence, wound healing problems, and pain of the sacroiliac joint [5]. Changing the position and orientation of the fixing screws is key to the success of the SAI technique; therefore, inspired by the SAI screw trajectory, we changed the traditional sacroiliac screw trajectory as follows: the sacroiliac screw started at the entry point (as shown in Fig. 1) on the dorsal side of the sacrum, passed through the sacral alar and sacroiliac joint, and extended into the iliac wing, thus we called it a “modified sacroiliac screw”.

The advantages of the modified sacroiliac screw over the traditional sacroiliac screw are reflected in the following aspects. First, after comparing screw trajectories, we concluded that the modified sacroiliac screws had a greater increase in screw length in the sacrum and iliac bone than traditional sacroiliac screws, thus the stability would be increased. Second, there were M2SI and M3SI screws in four modified sacroiliac screws passing through the intraosseous bone above the greater sciatic notch, thus the thread purchase force in the intraosseous of iliac will be further increased, and the stability and pullout resistance will be increased again (Note: The presence of the iliac grooves [25] that need special attention can affect the length of M2SI and M3SI screw placement, so preoperative three-dimensional pelvic model reconstruction is still necessary). Third, the direction of all modified sacroiliac screws is at an acute angle with the direction of the broken screw force, which will reduce the breakage rate of the modified sacroiliac screw. Fourth, based on the parameters of the modified sacroiliac screw that we measured, we chose the screw with the maximum radius, because the lateral bending strength of the screw is proportional to the radius to the 4th power. Fifth, modified sacroiliac screws can further stabilize the sacroiliac joint, thus significantly reducing lumbosacral pain caused by sacroiliac joint instability.

Due to the theoretical advantages of these modified sacroiliac screws, we conducted this study by measuring the parameters and angles of modified sacroiliac screws in order to instruct clinical application.

In view of the results, we found that each channel could be fitted with a screw of maximum radius and length. The screw radius decreased sequentially from M1SI to M4SI, and their mean radius ranged from 4.96 ± 0.38 mm to 3.4 ± 0.39 mm, while the gradual decrease in the volume of the sacrum from proximal to distal was responsible for tapering of the screw radius. The order of screw length was M4SI < M1SI < M2SI < M3SI, and the range of mean length of the screw was 89.05 ± 9.48 mm to 124.24 ± 12.64 mm. The trajectory of the M3SI screw started from the PSIS and extended to the ASIS (anterior superior iliac spine), and therefore, it was the longest screw. The range of the distance between the entry point and the PSIS was 25.29 ± 4.52 mm to 49.49 ± 7.43 mm. These data illustrated that the entry point for M2SI was closest to the PSIS, while M4SI was furthest from the entry point to the PSIS. As was summarized in the PSIS and *α* table, a number of statistically significant differences (*P* < 0.05) appeared in the genders, such as the PSIS length, while M4SI was furthest from the entry point to the PSIS. The mean screw radius of M3SI and M4SI screws were 4.26 ± 0.44 mm and 3.52 ± 0.32 mm for males and 3.91 ± 0.08 mm and 3.29 ± 0.42 mm for females. The mean screw length of M3SI and M4SI screws were 128.05 ± 8.15 mm and 91.39 ± 8.69 mm for males and 120.56 ± 15.45 mm and 88.63 ± 8.25 mm for females. The mean screw distance from the entry point to the PSIS of M3SI and M4SI screws were 34.58 ± 6.39 mm and 50.90 ± 7.49 mm for males and 33.39 ± 6.27 mm and 48.08 ± 7.18 mm for females. These data (*P* < 0.05) indicated that the modified sacroiliac screws used in men were thicker and longer than those used in women, and confirmed that the male pelvic wall was thicker and higher than the female pelvis. However, the *α* angle was greater in women than in men, since the mean screw *α* angle range of the M4SI screw was 32.99° ± 5.03° to 34.21° ± 4.98° for males and 34.98° ± 5.44° to 36.13° ± 4.56° for females. These data reminded us of the difference in the *α* angle in males and females during intraoperative screw placement and were also consistent with the characteristics of the female pelvis. There was no significant difference in the other parameters of the MSI screws, so we did not analyse and compare them, but they were equally important in guiding our clinical work.

Although we performed much work in this study, there were some limitations. First, the measurements of all data were manually measured on the real data pelvis simulated by the software, and there were artificial errors. Second, the pelvis model simulated by the software occurred after smooth operation, so there was a model distortion problem caused by the software, resulting in measurement error. Third, our study did not analyze the biomechanical aspects of the modified sacroiliac screws. Further research on modified sacroiliac screws is biomechanical research, especially the comparison between ordinary sacroiliac screws and modified sacroiliac biomechanics, so as to provide another reliable fixation method for clinical work.

## Conclusion

In summary, through the measurement of 100 healthy adult real three-dimensional pelvic models, we obtained the parameters of each screw and measured the three angles of each screw based on international coordinates for the first time, confirming the existence of modified sacroiliac screw channels. Preoperative three-dimensional reconstruction using Mimics and Three-matic software is very helpful for accurate screw placement. The theoretical advantages of modified sacroiliac screws over traditional sacroiliac screws indicate their great potential value. Further research should be done to show the potential clinical benefit of modified sacroiliac screws.

## Data Availability

The datasets generated and analysed during the current study are available from the corresponding author on reasonable request.

## Abbreviations

DICOM: Digital Imaging and Communications in Medicine
PSIS: The Posterior Superior Iliac Spine
ASIS: The Anterior Superior Iliac Spine
MSI screw: Modified Sacro-Iliac screw
M1SI: The first Modified Sacro-Iliac screw
M2SI: The second Modified Sacro-Iliac screw
M3SI: The third Modified Sacro-Iliac screw
M4SI: The fourth Modified Sacro-Iliac screw
R: The radius of modified sacroiliac screws
L: The length of modified sacroiliac screws
AP: The distance from the entry point to the posterior superior iliac spine
BS: The distance from the entry point to the median sacral crest
SAI srew: Sacral Alar-Iliac screw
S1AI: The first Sacral Alar-Iliac screw
S2AI: The second Sacral Alar-Iliac screw
S3AI: The third Sacral Alar-Iliac screw
IS screw: Iliac Screw
SI joint: Sacro-Iliac joint
CT: Computed Tomography
